# Olfactory sensory axons target specific protoglomeruli in the olfactory bulb of zebrafish

**DOI:** 10.1186/s13064-017-0095-0

**Published:** 2017-10-11

**Authors:** Xin Shao, Vanisha Lakhina, Puneet Dang, Ryan P. Cheng, Christina L. Marcaccio, Jonathan A. Raper

**Affiliations:** 1 Department of Neuroscience, University of Pennsylvania Perelman School of Medicine, Philadelphia, PA 19104 USA; 2 Department of Molecular Biology, Princeton University, Princeton, NJ 08540 USA; 3 105 Johnson Pavilion, 3610 Hamilton Walk, Philadelphia, PA 19104 USA

**Keywords:** Olfaction, Olfactory, Olfactory sensory neuron, Olfactory bulb, Axon guidance, OSN targeting, Odorant receptor, Odorant map, Protoglomerulus, Zebrafish

## Abstract

**Background:**

The axons of Olfactory Sensory Neurons (OSNs) project to reproducible target locations within the Olfactory Bulb (OB), converting odorant experience into a spatial map of neural activity. We characterized the initial targeting of OSN axons in the zebrafish, a model system suitable for studying axonal targeting early in development. In this system the initial targets of OSN axons are a small number of distinct, individually identifiable neuropilar regions called protoglomeruli. Previously, Olfactory Marker Protein-expressing and TRPC2-expressing classes of OSNs were shown to project to specific, non-overlapping sets of protoglomeruli, indicating that particular subsets of OSNs project to specific protoglomerular targets. We set out to map the relationship between the classical Odorant Receptor (OR) an OSN chooses to express and the protoglomerulus its axon targets.

**Methods:**

A panel of BACs were recombineered so that the axons of OSNs choosing to express modified ORs were fluorescently labeled. Axon projections were followed into the olfactory bulb to determine the protoglomeruli in which they terminated.

**Results:**

RNA-seq demonstrates that OSNs express a surprisingly wide variety of ORs and Trace Amine Associated Receptors (TAARs) very early when sensory axons are arriving in the bulb. Only a single OR is expressed in any given OSN even at these early developmental times. We used a BAC expression technique to map the trajectories of OSNs expressing specific odorant receptors. ORs can be divided into three clades based upon their sequence similarities. OSNs expressing ORs from two of these clades project to the CZ protoglomerulus, while OSNs expressing ORs from the third clade project to the DZ protoglomerulus. In contrast, OSNs expressing a particular TAAR project to multiple protoglomeruli. Neither OR choice nor axonal targeting are related to the position an OSN occupies within the olfactory pit.

**Conclusions:**

Our results demonstrate that it is not the choice of a particular OR, but of one from a category of ORs, that is related to initial OSN target location within the olfactory bulb. These choices are not related to OSN position within the olfactory epithelium.

## Background

Odorants are detected by dedicated odorant receptors deployed on the cilia of olfactory sensory neurons (OSNs) in the olfactory epithelium [1]. The axons of mature OSNs project from the olfactory epithelium to the olfactory bulb where they synapse with local and projection neurons within specialized neuropilar structures called glomeruli. OSNs that have chosen to express a particular main olfactory bulb type odorant receptor (OR) from a large OR gene repertoire extend axons to the same glomerular target, forming an OR-specific glomerulus [2, 3]. Odorants thereby induce neural activity in distinctive patterns of OR-specific glomeruli, effectively mapping odorant experience onto space in the olfactory bulb [4–6]. This mapping is thought to serve as the basis for odor discrimination [7, 8]. The organizing principles through which this mapping is established are therefore fundamental in understanding odorant perception.

Glomeruli form several days after sensory axons first reach the mouse olfactory bulb [9]. The glomerular locations for OSNs expressing approximately 20 of the more than 1000 ORs have been mapped in the mouse bulb. This was accomplished by either inserting sequences encoding histochemical tags into the genome so that they are transcribed along with specific ORs, or by *in situ* detection of odorant receptor mRNAs concentrated within sensory axons innervating glomeruli [2, 3, 10–18]. Glomeruli formed from the axons of OSNs expressing the same OR are reproducibly positioned within an area a few 100s of microns in diameter. OSNs that choose ORs within highly related subfamilies of clustered receptors project axons to glomeruli that are close to one another, although their relative positioning is variable [11, 19].

Our study was undertaken to better define the connections between OSNs that express particular odorant receptors and their very first targets in the olfactory bulb before OR-specific glomeruli form. We took advantage of the observation that in zebrafish, OSN axons extend directly into individually identifiable neuropilar regions called protoglomeruli [20]. Glomeruli are subsequently generated by the subdivision of protoglomeruli several days later in development [21]. Protoglomeruli can therefore be regarded as the initial targets for OSN axons. We used a BAC transgenesis-based approach to map the protoglomerular targets of OSN axons expressing 19 of the 143 ORs. This sampling represents 15 of the 37 OR subfamiles of clustered OR genes. Since ORs are expressed by the OMP-expressing class of OSNs [22], and since this class projects to the CZ, DZ, MG, and LG3 protoglomeruli [23], we expected to observe BAC labeled axonal projections to these four protoglomeruli. We hoped to determine if OSNs expressing particular ORs project to specific protoglomeruli within this subset, and if so, how OR expression is related to protoglomerular targeting.

We found that that OSNs expressing a particular OR projected to a single protoglomerulus. To our surprise, depending upon the OR expressed, OSNs projected to either the CZ or DZ protoglomeruli, and not to the MG or LG3 protoglomeruli. ORs can be divided into 3 broad clades on the basis of sequence similarity. OSNs that choose to express an OR from either of two clades project axons to the CZ, while those that express an OR from the third clade project to the DZ protoglomerulus. RNA-seq data from enriched OMP-class OSNs indicated that TAAR receptors are also expressed within the OMP class. The BAC expression approach was implemented for 3 TAARs. In each case OSNs that selected a particular TAAR receptor projected to all 4 OMP class protoglomeruli, suggesting the possibility that TAAR receptors represent major inputs for the MG and LG3 protoglomeruli. In sharp contrast to the mouse system [24], we found no relationship between the OR that an OSN chooses to express and its position within the olfactory epithelium. Thus, graded expression patterns of guidance receptors in the olfactory pit are unlikely to be translated into graded target positions within the olfactory bulb. Instead, it appears that OR choice is tightly coordinated with the expression of axon guidance cue receptors.

## Methods

### Transgenic zebrafish lines

Tg(omp:lyn-RFP) and Tg(trpc2:gap-Venus) transgenic lines (hereby referred to as TgOMP:RFP and TgTRPC2:Venus, respectively) were obtained from the Yoshihara laboratory [22]. Tg(UAS:gap43-Citrine), hereafter referred to as TgUAS:Citrine, was constructed as described in Lakhina et al. [23]. TgBACor111–7:IRES:Gal4 and TgBACor130–1:IRES:Gal4 transgenic lines were generated by injecting BACor111–7:IRES:Gal4 and BACor130–1:IRES:Gal4 into one cell stage embryos. All transgenic lines and wild type (Tupfel long fin) zebrafish were raised and maintained at 28.5 °C as described in Mullins et al. [25].

### RNASeq of OMP and TRPC2 neuronal populations

Single-cell suspensions of 48 hpf olfactory epithelia were FAC sorted to obtain RFP expressing or Venus expressing cells (RFP sample 1: 3500 cells, sample 2: 5500 cells; Venus sample 1: 676 cells, Venus sample 2: 201 cells). RNA was extracted using QIAGEN RNEasy kit and cDNA was synthesized using a custom oligo-dT primer containing a T7 promoter sequence courtesy of James Eberwine, University of Pennsylvania. In vitro RNA amplification was carried out through one round for OMP:RFP neurons and through two rounds for TRPC2:Venus neurons as described in Morris et al. [26]. Adapter-tagged libraries were synthesized using Illumina TruSeq v2.0 and deep-sequenced on HiSeq2500 to obtain ~100 million reads per sample. Reads were mapped to *Danio rerio* genome assembly GRCz10 using the STAR algorithm [27] and gene counts were generated using Verse [28].

### Plasmid construction

An IRES:Gal4-VP16 was cloned into the PWL82 vector (modified from PL451, Biological Resources Branch of Frederick National Laboratory for Cancer Research) kindly provided by Wenqin Luo with BamH1. Pcmlc2:GFP was cloned into iTol2 plasmid kindly provided by the Kawakami lab [29] with BglII. The OMP:Gal4 and UAS:Citrine plasmids were made by Lakhina et al. [23].

### BAC construction

CH211-24O8, CH211-222 M15, CH211-244E12, CH211-133D24, CH211-215A9, CH211-51 N5, CH211-199 M14, CH211-218 M13 were obtained from the BACPAC Resource Center (BPRC). DKEY-62A13 were obtained from Source BioScience. The SW105 bacterial strain (Biological Resources Branch of Frederick National Laboratory for Cancer Research) was transformed with BACs of interest, shaken in a 42 °C water bath for 15 min, and then chilled on ice for 20 min to induce λRed recombinase. PCR products containing insertion cassettes were electroporated into competent SW105 bacteria and integrated into the BAC by recombination [30, 31]. IRES:Gal4-VP16 and a neo selection cassette flanked by 50 bp homology arms targeted insertion immediately after the stop codon of selected odorant receptors. Pcmlc2:GFP and a neo selection cassette flanked by inverted mini-tol2 arms and nested in 50 bp homology arms induced insertion at a site distant from the OR gene cluster. After insertion, 10% L-arabinose was added to induce Flp recombinase and the deletion of the Neo cassette. The insertion of Gal4 and GFP constructs by recombination were performed sequentially. The iTol2 sequence facilitates BAC integration when injected into just fertilized eggs along with the Tol2 recombinase [29]. Pcmlc2-GFP was used to identify transfected and transgenic embryos containing the BAC by fluorescent imaging of whole embryos, and the IRES:Gal4 was used to visualize olfactory sensory neurons that chose to express odorant receptors from recombineered BACs.

### Microinjections into zebrafish embryos

Microinjections of purified BAC (NucleoBond® BAC 100 from Clontech) or plasmid constructs (GeneJet Plasmid Maxiprep Kit from Fermentas Life Sciences) into zebrafish embryos were performed as described in Fisher et al. [32]. Approximately 2 pgm OMP:Gal4 and/or UAS:Citrine containing plasmids were injected along with 35 pgm RNA for mini-Tol2 recombinase into one cell stage zebrafish embryos. Approximately 50 pgm of BAC was similarly injected along with 50 pgm RNA for mini-Tol2 recombinase as described in [29].

### In situ probe construction, hybridization, and fluorescent visualization

RNA was extracted from the heads of 3 dpf wild type zebrafish and used to perform RT-PCR with degenerate or specific OR primers that amplified ORs belonging to the OR111, OR106, OR128, OR133, OR125, or OR103 subfamilies. PCR products (~ 650 bp long) were cloned into the pCR II-TOPO vector and recovered OR sequences were identified by sequencing. Digoxigenin and fluorescein labeled antisense RNA probes were generated from OR-containing plasmids by in vitro transcription (T7 and Sp6 from Promega Corp.). Embryos were incubated in 0.2% DEPC-Collagenase 25 °C for 2 h, hybridized with OR probes in DEPC-HYB+ and in situ signals were amplified using a Cyanine 3-coupled tyramide signal amplification system (TSA Plus Cyanine 3 System, Perkin Elmer, Product number: NEL744001KT), a Cyanine 5-coupled tyramide system (TSA Plus Cyanine 5 System, Perkin Elmer, Product number NEL745001KT) or a Fluorescein-coupled tyramide system (TSA Plus Fluorescien System, Perkin Elmer, Product number NEL741001KT) as described in Chalasani et al. [33].

### Immunohistochemistry

Zebrafish embryos were fixed in 4% paraformaldehyde in 0.1 M phosphate buffer overnight at 4 °C. They were permeabilized by treatment with 0.1% DEPC-Collagenase 25 °C for 2 h, washed in incubation buffer (0.2% BSA, 0.5% Triton-100 in 0.1 M phosphate buffer), incubated in Goat anti-GFP (1:100, Rockland Immunochemicals, catalog #600–101-215) overnight at 4 °C, and then in anti-goat IgG Alexa Fluor 488 (1:500; Invitrogen, catalog#A11055) overnight at 4 °C. All incubations in antibody were performed in Incubation buffer. Propidium iodide staining was performed as described in Brend et al. [34] with the omission of the RNase treatment step.

### Imaging and analysis

Embryos were mounted in Vectashield Mounting Media in a frontal orientation and imaged using a 40X or 60X oil immersion lens on a Leica TSP5 confocal microscope. Confocal sections were collected at 1 μm intervals from anterior to posterior from the olfactory pit through the olfactory bulb. The number of OBs with axons terminating in particular protoglomeruli and the number of neurons with in situ signal in each OB were counted. Analyzed fish were of indeterminate sex at the stage we examined (3 dpf). Fisher’s two-tailed exact test was used to estimate statistical significance.

### OR expression mapping

The relative locations of all OSN cell bodies expressing a particular OR or TAAR were visualized by confocal microscopy and recorded in z-stacks of images. Stacks from pits on one side of the larvae were reversed to that all images had the same orientation. Reference structures in the stacks were chosen as fiduciary marks and included the nearest neuromast (NM) and the center of the olfactory pit (CP). The NM and the CP were recorded as single points. The pit is tear-shaped in cross section and the dorsal vertex was used to define a reference axis specified by three points taken at 5 um intervals along the anterior-posterior axis. A best-fit line was generated for these 3 vertex points. OR maps were overlaid on a virtual coordinate system for comparison as follows. Coordinates representing OR expression maps and reference structures were adjusted by translation so that they were centered on CP, and then rotated in 3 dimensions so that the line defined by the vertices aligned. The positions of NMs were used to confirm that the maps were aligned and correctly merged.

### Statistical methods

The numbers of OBs with axons in particular protoglomerui were tabulated and Fisher’s exact test (two-tailed) was used to determine statistical significance (Figs. 5 and 7).

## Results

### OMP class OSNs project to a limited number of specific identifiable protoglomeruli

**Fig. 1:**
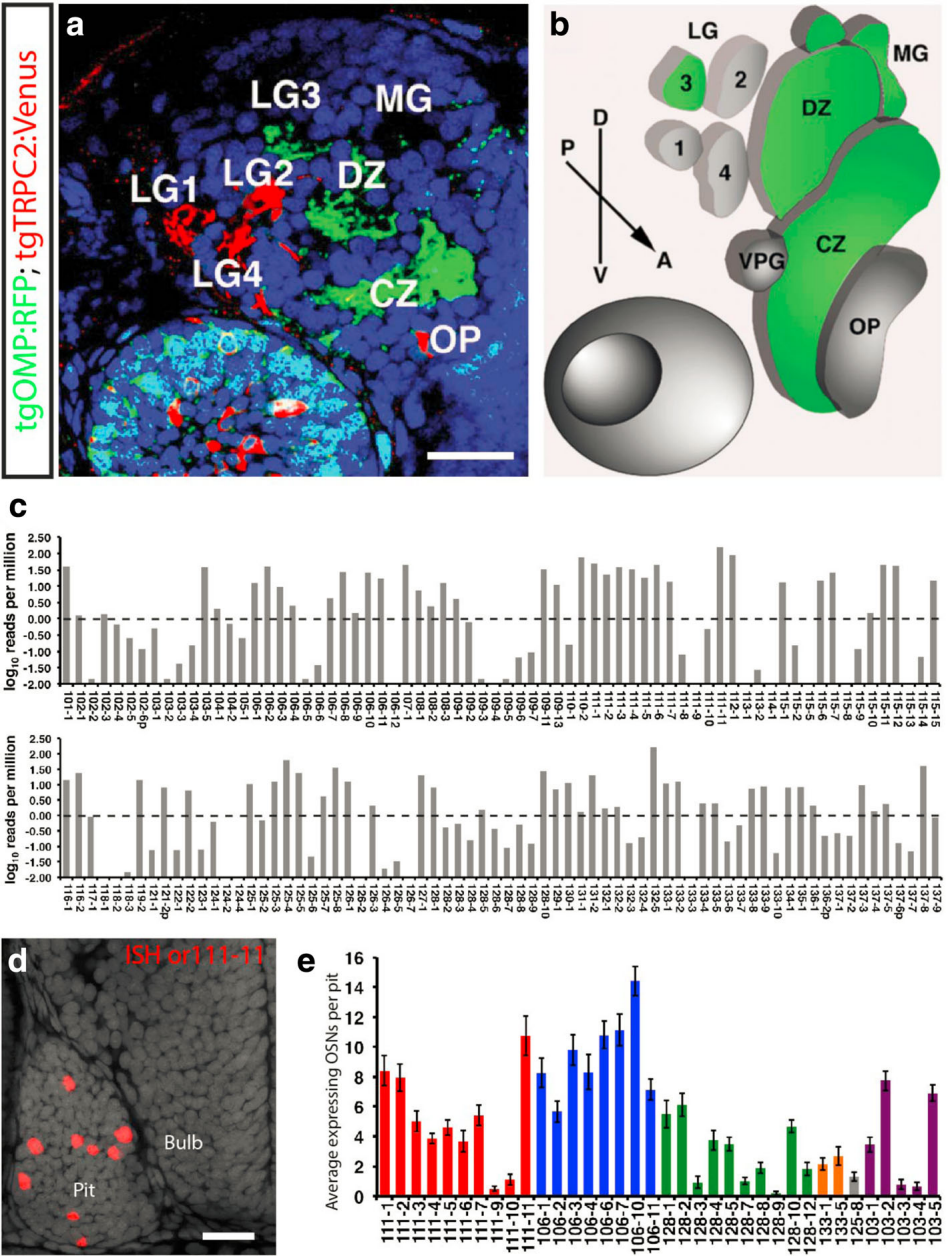
OMP class OSNs project to a limited number of identifiable protoglomeruli but express a wide variety of ORs. **a** A confocal optical section through the olfactory bulb of a double transgenic 3 dpf embryo showing that OMP class ciliated (green) and TRPC2 class microvillous (red) sensory neurons target non-overlapping protoglomeruli. **b** OMP class neurons innervate the CZ, DZ, LG3, and to a lesser extent, MG protoglomeruli. **c** RNA-seq data from 2 dpf FAC sorted and pooled OMP:RFP expressing OSNs. Approximately ½ of the OR gene repertoire is expressed at an average level of over 1 cpm in the pool as a whole (dashed line). **d** Expression of *or111–11* as visualized by *in situ* hybridization of its mRNA in a 3 dpf embryo. **e** The average numbers of OSNs expressing selected ORs at 3 dpf. Scale bars: 20 μm

There are three broad classes of OSNs that can be identified by their morphology in the zebrafish olfactory epithelium. Ciliated olfactory sensory neurons express main olfactory bulb type ORs along with the Olfactory Marker Protein OMP. Microvillous sensory neurons express V2R-type odorant receptors along with the TRPC2 channel [22]. Crypt class sensory neurons express the V1R type receptor ora4 [35]. Another more poorly characterized class of sensory neurons expresses members of the TAAR family of Trace Amine Associated Receptors [36]. Transgenes for OMP:RFP (immunolabeled green) and TRPC2:Venus (immunolabeled red) label olfactory sensory axon projections from the first two classes of OSNs (Fig. 1a). The axons of each class target a complementary, non-overlapping, and individually identifiable subset of protoglomeruli in the bulb [22, 23]. OMP expressing sensory neurons project to the Central Zone (CZ), Dorsal Zone (DZ), Lateral 3 (LG3), and to a lesser extent, the Medial Glomeruli (MG) protoglomeruli at 3 dpf (green shaded areas Fig. 1b). We set out to determine whether OMP class OSNs that express particular ORs project to specific protoglomeruli within this set. We first characterized the ORs expressed early in development close to the time when OSN axons first reach the olfactory bulb.

### Many odorant receptors from multiple subfamilies are expressed in OSNs early in development

An RNA-seq approach was used to determine how many of the 143 main olfactory bulb type ORs are expressed in 2 dpf embryos, just after OSNs have first entered the olfactory bulb [20, 37]. OMP:RFP transgene-expressing OSNs were isolated by FACS and cDNA libraries were produced from pooled OMP or TRPC2 class neurons. Two independent libraries were sequenced for each class to a depth of approximately 100 million reads and were mapped onto the GRCz10 zebrafish genomic scaffold [38]. Even at this early stage, sequences corresponding to a large proportion of the OR gene repertoire were detected to varying degrees (Fig. 1c). As the cpm represent an average of all the OMP class neurons from which the libraries were made, the level at which an individual OSN expresses its chosen OR would be expected to be roughly 100 fold higher than the measured average.

Our data suggest that a large proportion of the OR repertoire is expressed very early, but it is difficult to determine the frequency at which OSNs express particular ORs from the RNA-seq data. We performed *in situ* hybridization studies to determine how many sensory neurons express particular selected ORs. RT-PCR with combinations of degenerate primers corresponding to specific subfamilies of odorant receptors, or specific primers targeting individual odorant receptors, were used to recover the full coding sequences of over 30 individual ORs. We obtained sequences for nearly all of the odorant receptors for which we designed primers from cDNA prepared from 3 dpf embryos, consistent with the idea that a large proportion of the overall OR repertoire is expressed early. These sequences were used to generate probes for *in situ* hybridization to visualize OR expression in 3 dpf olfactory pits (Fig. 1d). The average number of OSNs expressing each different family member from several different subfamilies were determined (Fig. 1e). The number of OSNs per pit expressing a given receptor ranged from ~1 or less (*or111–9, or111–10, or128–3, or128–7, or128–9, or103–3*, and *or103–4*) to ~10 or more (*or111–11, or106–3, or106–6, or106–7, or106–10*). Large differences in the numbers of expressing sensory cells were observed even within the same subfamily of receptors.

### Each olfactory sensory neuron expresses a single or a very limited number of odorant receptors at 3 dpf

**Fig. 2:**
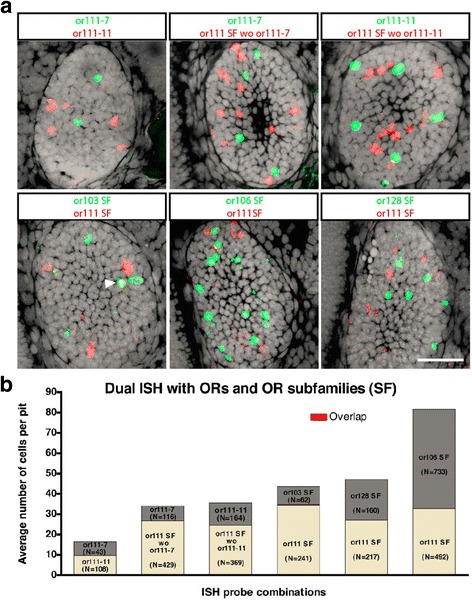
Dual *in situ* hybridization with probes to multiple ORs are consistent with OSNs having chosen a single odorant receptor to express by 3 dpf. **a** Examples of pairs of probes or cocktails of probes used to access OR expression in 3 dpf embryos. The first row of panels demonstrates that probes against individual or111 subfamily members *or111–7* and *or111–11* are highly specific to the target subfamily member, and further suggest, that individual or111 subfamily members are very rarely, if ever, co-expressed in the same OSN. The second row of panels demonstrates that or111 subfamily members are very rarely co-expressed in OSNs expressing *or103*, *or106*, or *or128* subfamily members. An exceptional case where *or111* and *or103* subfamily members were coexpressed is marked with an arrowhead. **b** Quantification of the numbers of probed OR expressing OSNs per olfactory pit. Cells with overlapping expression are indicated (red). Scale bar: 25 μm

To determine whether sensory neurons express a single odorant receptor as early as 3 dpf, we performed two-color *in situ* hybridization on zebrafish olfactory epithelia with several combinations of odorant receptor specific probes. These included probes for or111–7 and or111–11; or111–7 and a cocktail of probes for all of the other or111 family members; or111–11 and a cocktail of probes for all of the other or111 family members; cocktails of all OR111 family members and OR103 family members; cocktails of all OR111 family members and OR106 family members; and cocktails of all OR111 family members and OR128 family members (Fig. 2a). The results of these observations are quantified in Fig. 2b. There is almost no overlap in the expression patterns observed between any of these probes or groups of probes. This is true even when the expression of closely related receptors within the OR111 subfamily are compared. These results demonstrate the high specificity of the *in situ* probes and supports the idea that by 3 dpf an OSN expresses only one, or at most a very small number, of ORs.

That so many different ORs are expressed at this developmental time, that each OSN expresses only a single OR, and that the OMP and OR expressing OSNs project to only 4 protoglomeruli, necessarily requires that a single protoglomerulus is targeted by OSNs that collectively express many different ORs. What determines which protoglomerulus an OSN targets? Is targeting related to OR identity? Do OSNs that express highly related ORs target the same protoglomerulus? These questions prompted us to ask whether OSNs that choose to express highly related ORs from the same subfamily of OR receptors target the same or different protoglomeruli.

### Olfactory sensory neurons expressing odorant receptors from the endogenous OR111 subfamily gene cluster target the central zone protoglomerulus

**Fig. 3:**
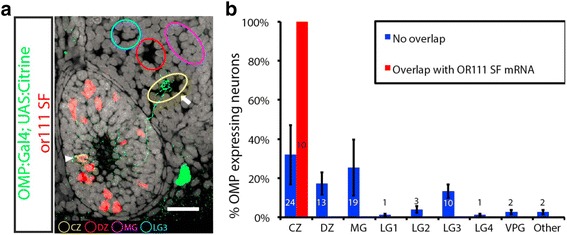
Mapping axon projections by sparse labeling of OMP class OSNs. **a** A single OSN in a 3 dpf embryo was co-labeled by transient transfection with a combination of OMP:Gal4 and UAS:Citrine plasmids and hybridization with a cocktail of or111 subfamily in situ probes (arrow head). The CZ protoglomerulus is visible as a cell free zone in the olfactory bulb (arrow). **b** Single OMP:Gal4;UAS:Citrine labeled OSNs project to the CZ, DZ, LG3, and MG protoglomeruli (blue bars). Those that are co-labeled with a cocktail of or111 subfamily probes project exclusively to the CZ protoglomeruls (red bars). The numbers of cells observed for each condition are noted within the bars. SEM is indicated. Scale bar: 20 μm

To test whether all members of the or111 subfamily target the same protoglomerulus, just-fertilized embryos were transiently transfected by injection with two plasmids encoding Gal4 driven by the OMP promoter (OMP:Gal4) and a Gap43-Citrine fusion protein driven by a Gal4 activated promoter sequence (UAS:Citrine). Low concentrations of plasmid were used to ensure that only about one sensory neuron expressed Citrine in each olfactory pit. At 3 dpf, transfected embryos were collected and reacted with a cocktail of in situ probes corresponding to most members of the or111 subfamily. Olfactory pits in which only a single sensory neuron was fluorescently labeled with Citrine were identified and analyzed. In most instances, the single fluorescent sensory neuron was not found to express a member of the or111 subfamily. Each of these non co-labeled sensory neurons projected a single axon to any one of the four protoglomeruli that normally receive innervation from the OMP class of sensory neurons: the CZ, DZ, MG, or LG3 protoglomeruli (blue bars in Fig. 3b). Occasionally, a single fluorescent sensory neuron was found to express a member of the or111 subfamily (Fig. 3a). In these instances, its axon always projected to the CZ protoglomerulus (Red bar in Fig. 3b). If the probability that a randomly labeled sensory neuron projects to the CZ protoglomerulus is estimated accurately by the percentage of randomly labeled GFP expressing neurons as a group, then the probability that all Citrine and or111 co-labeled cells projected to the CZ by chance is p < =0.0001. These results are consistent with the idea that sensory neurons expressing any member of the or111 subfamily project to the CZ protoglomerulus. They support the conclusion that OSNs choosing to express a particular OR project to a single specific protoglomerulus, and they further suggest that OSNs expressing highly related ORs project to the same protoglomerulus.

### OSNs expressing particular ORs target specific, single protoglomeruli

**Fig. 4:**
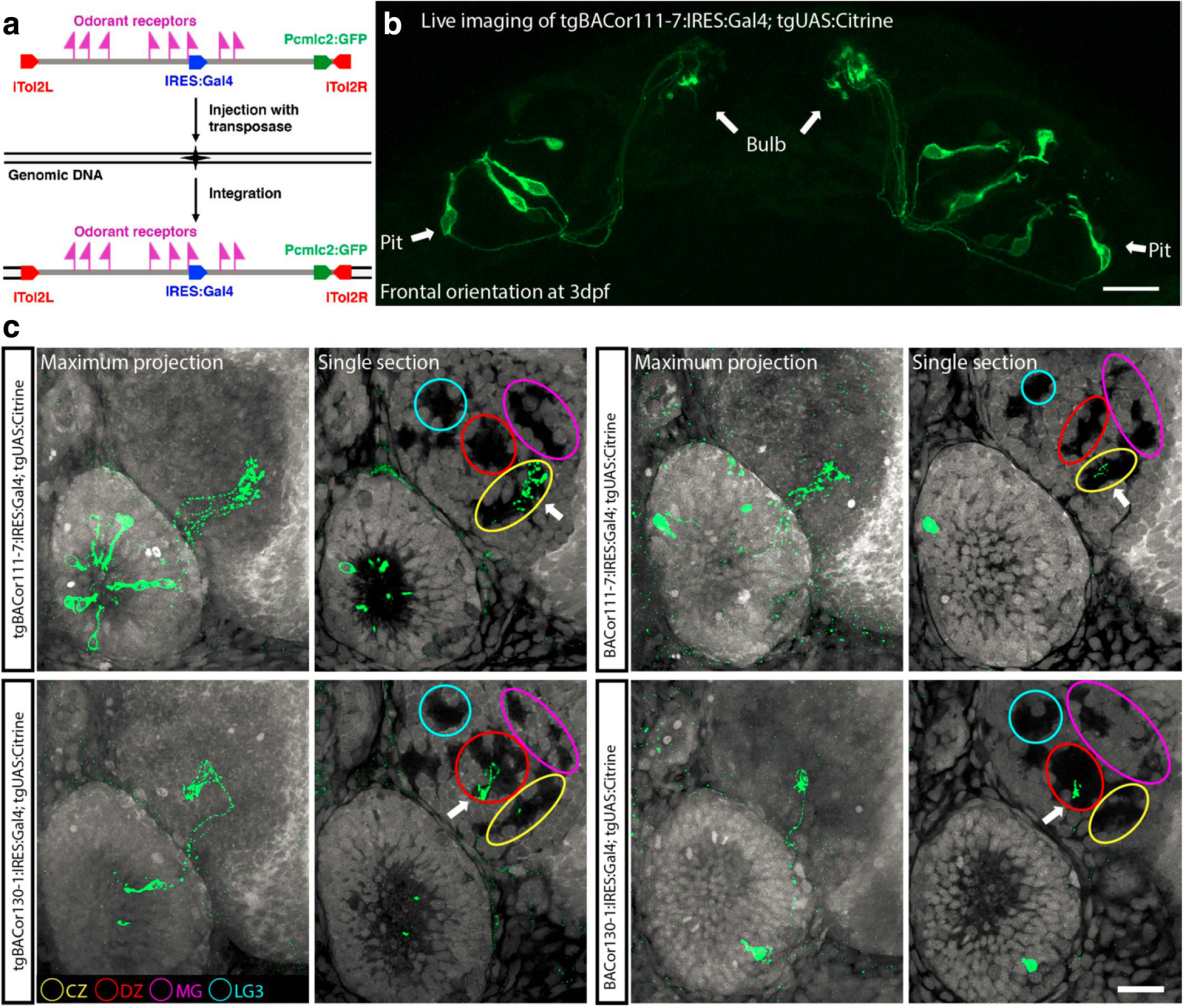
Mapping OSN axon projections with recombineered BACs. **a** IRES:Gal4 was inserted just after the coding sequences of selected ORs and a heart-specific promoter driving fluorescent GFP was inserted at a second location distant from the co-labeled OR (see text and Methods for details). **b** Live confocal image from a double transgenic embryo in which *or111–7* driven Gal4 activates UAS:Gap43-Citirine. A small number of OSNs are labeled with Citrine allowing the visualization of their cell bodies in the olfactory pits, axonal projections, and sites of termination in the olfactory bulb. **c** OSN projections in embryos from tgBAC*or111–7*:IRES:Gal4x tgUAS:Citrine or tgBAC*or130*–1:IRES:Gal4 x tgUAS:Citrine lines (left two columns). The same axonal projection patterns are observed in UAS:Citrine embryos transiently transfected with the BACs used to make the transgenic lines (right two columns). 3 dpf fixed embryos were co-stained with anti-GFP (green) and Propidium Iodide (Grey). The CZ (yellow), DZ (red), LG3 (blue), and MG (magenta) protoglomeruli are indicated on each single optical section. *or111–7* expressing OSNs project to the CZ protoglomerulus, while *or130–1* expressing OSNs project to the DZ protoglomerulus. Scale bar: 20 μm

We used BAC recombineering [30, 31] and BAC transgenesis to map the initial target locations of OSNs expressing particular ORs. BACs were selected that spanned at least one entire OR subfamily cluster. An IRES driving the yeast transcription factor Gal4 was recombined just after the coding sequence of a selected OR (Fig. 4a). Thus, mRNA including both the selected OR and IRES:Gal4 are transcribed when that particular OR is chosen for expression. Gal4 subsequently activates the expression of UAS:Citrine which labels the cell bodies and axonal processes of expressing sensory neurons. Each BAC was engineered through a second recombination to include inverted tol2 sites adjacent to a heart specific promoter driving GFP expression [39]. These sequences were inserted at a large distance from the modified OR locus. BACs containing modified OR loci containing IRES:Gal4 were repeatedly injected into just-fertilized UAS:Citrine transgene containing eggs along with tol2 transposase to facilitate the incorporation of BAC DNA into random genomic loci [29]. Injected embryos were harvested 3 days later, those with GFP labeled hearts were selected, and processed with anti-GFP to further enhance the OR-specific Citrine signal. Permanent transgenic lines were made with two BACs: tgBACor111–7:IRES:Gal4 and tgBACor130–1:IRES:Gal4. OSNs which chose to express the OR co-labeled with Gal4 in a living 3 dpf tgBACor111–7:IRES:Gal4;UAS:Citrine embryo are visualized by confocal microscopy in Fig. 4b. The sensory cell bodies, their axons, and their terminations in the bulb are evident. Axonal targeting was examined in fixed 3 dpf embryos produced from either transgenic fish lines or transient transfections made with BACs (Fig. 4c). Counterstaining with propidium iodide allows the visualization of cell free protoglomeruli. OSNs expressing or111–7 had axons projecting to the CZ protoglomerulus. In contrast, OSNs expressing or130–1 had axons projecting to the DZ protoglomerulus. OSNs labeled by transient BAC transfections projected to the same protoglomeruli as those labeled in the permanent transgenic lines.

**Fig. 5:**
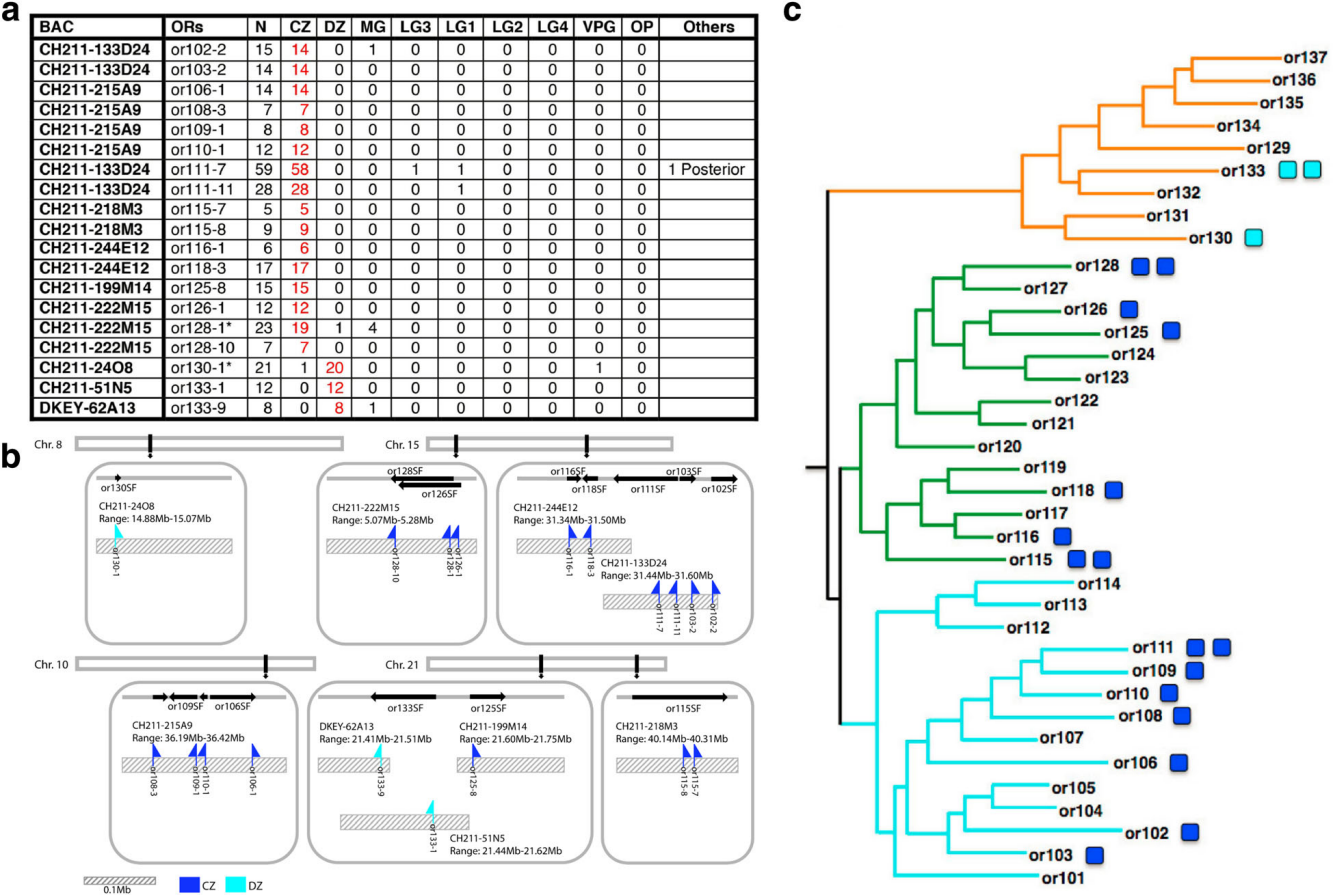
OSNs that choose to express a particular OR project to a specific protoglomerulus. **a** Cumulative protoglomerular projection data for each of the ORs examined in this study. BAC: BAC employed, ORs: specific OR modified with IRES:Gal4 just after its coding sequence, N: total number of olfactory pits with labeled sensory neurons observed. The remaining columns tabulate the number of pits with axons projecting to the indicated protoglomeruli. If a pit had sensory axons projecting to more than one protoglomerulus, the number of counts can exceed N. The predominantly targeted protoglomerulus is indicated in red. **b** The relationships between OR, BAC, and genomic positions for the BACs used in this study. Chromosomes are pictured at the top with the approximate locations of BACs in black. Within each box OR gene clusters are represented with black arrows showing the direction in which genes are transcribed. BACs employed are represented by hashed bars and the approximate locations of each of the ORs we co-labeled with IRES:Gal4 are indicated with flags. Projection to the CZ (dark blue) or the DZ (light blue) is indicated. Scale bar: 0.1 Mb. **c** OSNs that chose to express ORs from clade A comprising subfamilies or101–114, or clade B comprising subfamilies or114–128, were found to project to the CZ protoglomerulus (dark blue boxes). OMP class OSNs that chose to express members of clade C comprising subfamilies or129–137 project to the DZ protoglomerulus (light blue boxes)

Additional BAC constructs with modified ORs were transiently expressed in UAS:Citrine embryos and their projections examined at 3 dpf (Fig. 5a). Data are reported for 9 separate BACs with a total of 19 modified ORs. This sampling represents 19/143 (~13%) of all OR genes, 15/37 (40%) of all OR gene subfamily clusters encoding 99/143 (~70%) ORs, and 6 of the 10 OR superclusters encoding a total of 124/143 (~85%) ORs. OSNs expressing each one of these ORs were found to project axons almost exclusively to the same specific protoglomerulus (Fig. 5a). A total of 16 projected to the CZ protoglomerulus, while 3 projected to the DZ protoglomerulus.

These findings suggest that: [1] OSNs expressing Main Olfactory Bulb type ORs project to only the CZ and DZ protoglomeruli, and [2] the particular OR that is expressed is related to whether an OSN specifically targets the CZ or the DZ protoglomerulus. ORs associated with CZ projections come from many different subfamily clusters on many different chromosomes (Fig. 5b), and although we have fewer instances from which to generalize, the same pattern seems to hold true for OSNs projecting to the DZ. Further, our results are consistent with a model in which OSNs expressing any member of the same OR subfamily project to the same protoglomerulus. Unexpectedly, we found that CZ targeting was associated with the expression of ORs from either of two OR clades, while DZ targeting was associated with expression from the third remaining OR clade (Fig. 5c). These results strongly suggest that guidance receptor choice and OR subfamily choice are tightly linked.

### Sensory neurons that express particular selected TAARs project to multiple protoglomeruli innervated by OMP class OSNs

**Fig. 6:**
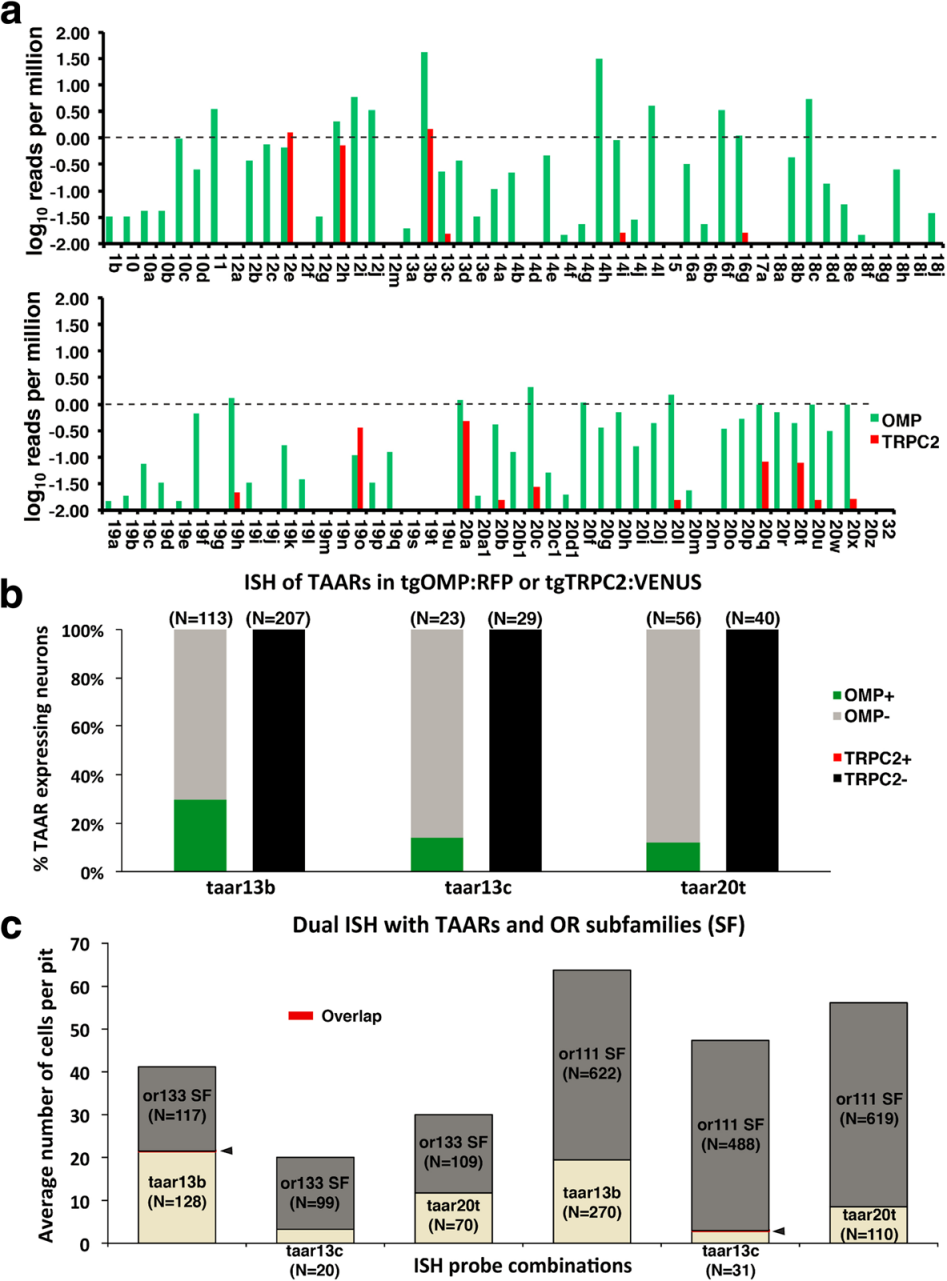
Some OMP class OSNs express TAARs instead of ORs. **a** TAAR expression profiles were generated from pools of FAC sorted OMP or TRPC2 class OSNs collected from 2 dpf OMP:Venus or TRPC2:RFP embryos. The dashed line demarcates the 1 cpm expression level in the pool as a whole. A wide variety of TAAR receptors were more prominently expressed in OMP as compared to TRPC2 class neurons. **b** A portion of OSNs labeled by *in situ* probes for each of the three TAAR receptors overlapped with OMP transgene expressing cells at 3 dpf. None of the TAAR expressing OSNs overlapped with TRPC2 transgene expressing cells. **c** OR and TAAR expression are mutually exclusive at 3 dpf. Overlapped expression (arrowheads) was extremely rare in double *in situs* with TAAR probes co-labeled with cocktails of either or111 or or133 subfamily probes

We were surprised that none of the OSNs expressing any of the 19 Gal4-labeled OR genes projected axons to the OMP positive protoglomeruli LG3 or MG. It is possible that OSNs expressing some of the main olfactory bulb OR subfamilies that we did not test project to these targets. Another possibility is that these protoglomeruli are targeted by OSNs expressing the TAAR class of odorant receptors. Previous work reported that* taar13c* is expressed in ciliated OMP-expressing OSNs [40], suggesting that OMP class OSNs could express either ORs or TAARs. We performed RNA-seq analysis of FAC sorted pools of OMP:RFP or TRPC2:Venus OSNs from 2 dpf olfactory pits. TAAR expression is much more pronounced in OMP as compared to TRPC2 positive cells (Fig. 6a). We infer that some portion of OMP class sensory neurons express TAARs, and hypothesized they project to OMP positive protoglomeruli.

As a first test of this idea we reacted transgeneic OMP:RFP or TRPC2:Venus embryos with probes for each of three selected TAARs: *taar13b*, *taar13c*, or *taar20t*. In each case, between approximately 10–30% of TAAR expressing cells overlapped with OSNs labeled with RFP in a transgenic line in which RFP is driven by an OMP promoter (Fig. 6b). No overlap was observed with OSNs labeled with Venus in a transgenic line in which Venus is driven by a TRPC2 promoter. These results confirmed the RNA-seq results suggesting that at least some TAAR expressing OSNs overlap with the OMP class as defined by the transgenic line we are using.

We next asked whether ORs and TAARs can be co-expressed in OSNs. Cocktails of probes for either the or111 or the or133 subfamilies were paired with probes for each of the 3 TAARs. In all six combinations, essentially no overlap between OR and TAAR probes was observed (Fig. 6c).

**Fig. 7:**
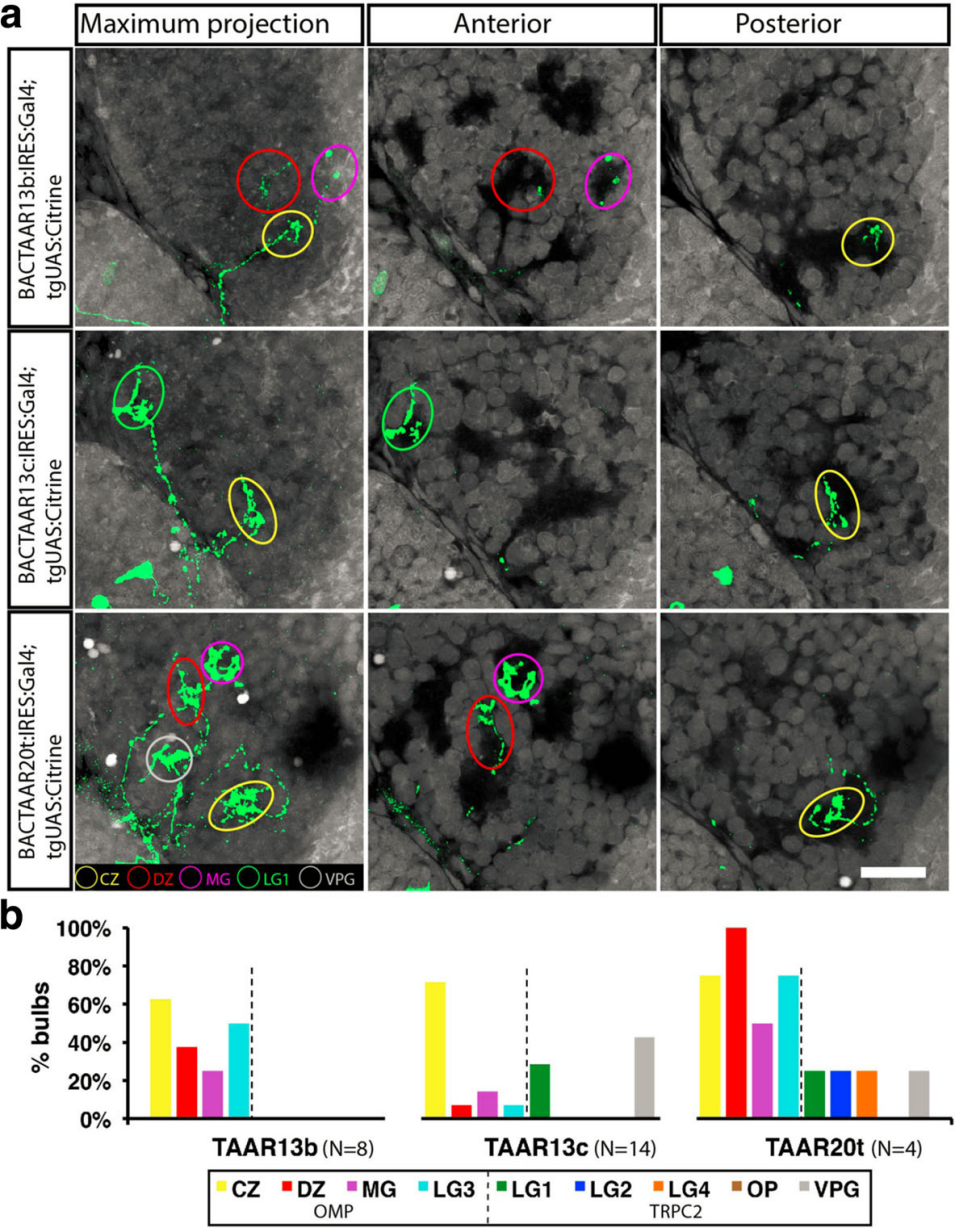
**a** OSNs expressing Gal4 from each of three selected TAAR loci all project to multiple protoglomeruli. **b** OSNs labeled by expression from the *taar13b* locus projected to all four OMP class protoglomeruli, while OSNs expressing from the *taar13c* or *taar20t* loci projected to both OMP and TRPC class protoglomeruli. Scale bar: 20 μm

To determine where OSNs that express TAARs project in the OB, we generated three BAC constructs in which IRES:Gal4 was co-transcribed with *taar13b*, *taar13c*, or *taar20t*. Transient expression experiments with these BACs (Fig. 7a) showed that, in aggregate, OSNs expressing* taar13b* project axons to all of the OMP positive protoglomeruli: CZ, DZ, LG3, or MG (Fig. 7b). Similar results were obtained with OSNs expressing *taar13c*, although some of these cells also had projections to the TRPC2 positive LG1 and VPG protoglomeruli. OSNs expressing *taar20t* projected to all OMP and many TRPC2 positive protoglomeruli.

Our results are consistent with the hypothesis that at least some TAAR expressing OSNs are members of the OMP class. At least at this early stage of development, TAAR expressing OSNs likely target all OMP positive protoglomeruli and possibly TRPC2 positive protoglomeruli as well. Most importantly, in distinct contrast to OSNs expressing main olfactory bulb type ORs, sensory neurons that choose to express the same TAAR receptor can target multiple protoglomeruli. Assuming our BAC based approach provides an accurate measure of OSN targeting, TAAR based olfactory circuitry appears to be organized during development in a fundamentally different way than OR based circuitry.

### OR expression is unrelated to OSN position within the olfactory epithelium

**Fig. 8:**
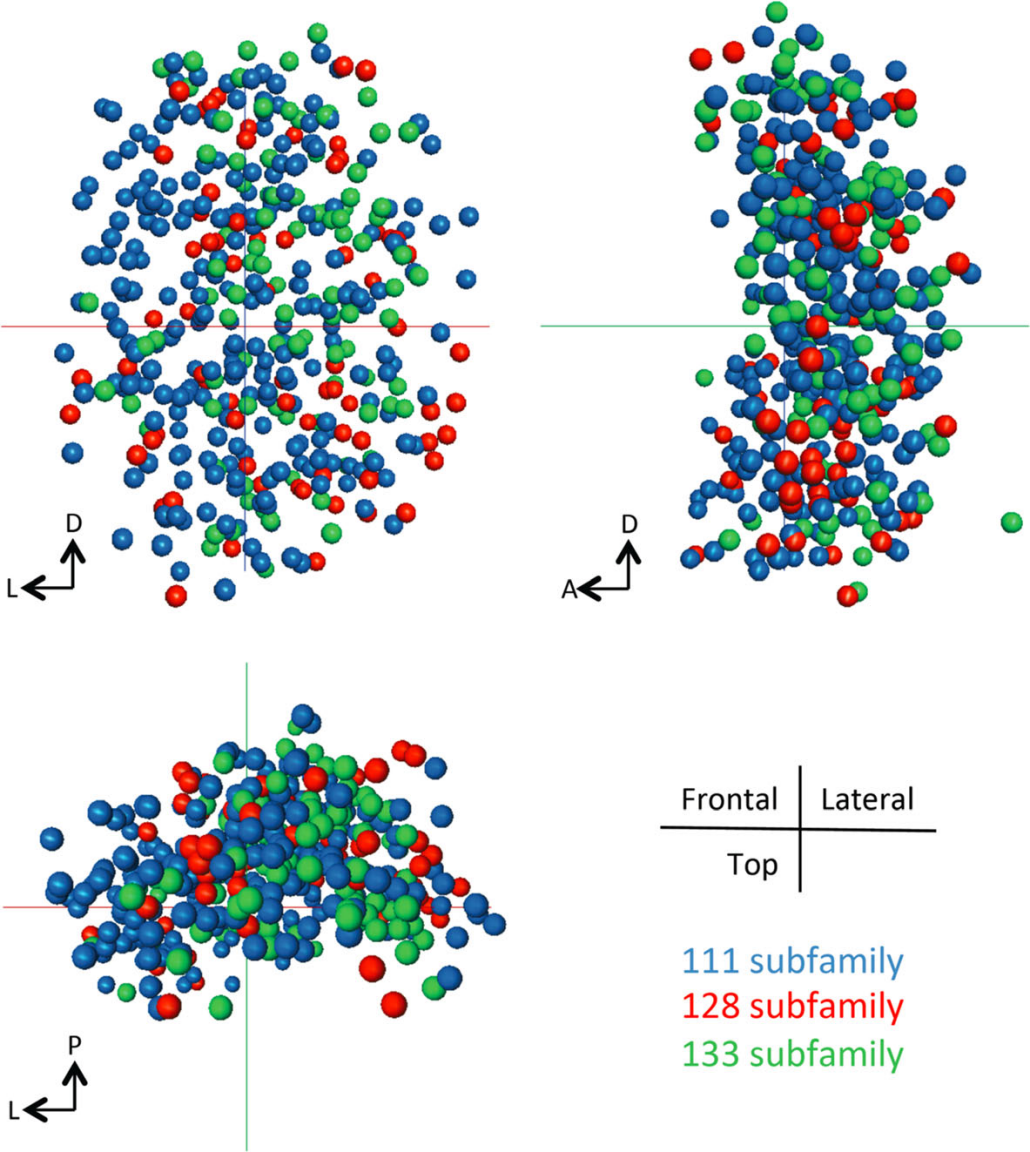
OSNs expressing odorant receptors from each of the three OR clades are intermixed within the olfactory epithelium. Cocktails for the or111 (clade A), the or128 (clade B), or or133 (clade C) subfamilies were used to probe multiple 3 dpf olfactory pits (5 olfactory pits for each subfamily). The positions of each labeled OSN were recorded, aligned using common reference points, and then projected onto the same spatial coordinates. OSNs expressing each of the 3 OR subfamilies are found medially, laterally, dorsally, and ventrally. No regionalized expression is apparent for any of the three, and they appear to be intermixed in Frontal, Lateral, or Horizontal views

The clade from which an OSN chooses an OR and the initial target location of its axon are closely related. Could these in turn be related to OSN position in the olfactory epithelium? To address this question we overlaid the position of OSNs expressing members of the or111 (clade A), the or128 (clade B), or or133 (clade C) subfamilies onto a single virtual olfactory pit to compare their relative distributions (Fig. 8). Probes for each subfamily label OSNs in the dorsal, the ventral, the medial, and the lateral regions of the pit. We did not detect any strong regionalization in the expression of any single ORs or OR subfamilies.

## Discussion

### Odorant receptor choice occurs early

ORs are first expressed very early in zebrafish development while OSN axons are extending into the OB [41–43]. Previous *in situ* studies in mature fish with dual OR probes support the idea that an OSN chooses a single OR to express [42], although Sato [44] reported that OSNs can co-express some members of the or103 subfamily. We have now performed an extensive series of dual in situ experiments to access whether OSNs can express more than one OR early in development as their axons are extending to the OB. In multiple combinations of probes for single ORs, or cocktails of OR subfamily members, we observed essentially no co-expression of ORs in OSNs of 3 dpf fish. In a separate single cell RNA-seq study of OSNs isolated at 2 dpf, a day earlier than those employed for the in situ studies reported here, we found that most of the OSNs we collected expressed a single predominant OR (Dang et al., unpublished). Our findings are consistent with observations using single cell RNA-seq of OSNs isolated from early postnatal mice. The expression of a single predominant OR was demonstrated once an OSN reaches a certain level of maturity [45, 46]. Less mature OSNs were observed to express more than one OR at significant levels. Taken together, these findings suggest that most OSNs have made stable and exclusive odorant receptor choices even at very early developmental stages when axons are extending to the olfactory bulb, but they fall short of demonstrating that OR choice is complete before OSN axons reach or select their targets within the bulb.

### Odorant receptor choice and protoglomerular targeting are coordinated

Using a BAC expression approach we were able to map the connectivity between OSNs expressing particular ORs to their initial targets within the olfactory bulb. This was possible since, as first noted by Dynes and Ngai [20], OSN axons in the zebrafish extend directly from the olfactory epithelium to distinct, individually identifiable neuropil regions called protoglomeruli. Sato et al. [22] showed that specific protoglomeruli are innervated by OMP expressing ciliated OSNs, while a complementary set of glomeruli are innervated by TRPC2 expressing microvillous OSNs. These findings demonstrated that protoglomeruli receive class-specific innervation. In this study we found that within the OMP class of OSNs, protoglomerular targeting is tightly linked to the OR a cell chooses to express.

We inserted IRES:Gal4 behind the coding sequences of selected ORs in BACs that contained clusters of OR genes. We reasoned that BACs contain sufficient promoter and enhancer elements to provide a reasonably accurate reflection of OR expression in vivo. One advantage of the BAC approach is that a large number of constructs could be recombineered, injected into just-fertilized eggs, and just a few days later, labeled axonal projection patterns assessed. A wide variety of ORs were examined from ~40% of all OR subfamilies and ~13% of the total OR gene repertoire [47]. It is worth pointing out that for any given OR, our results were accumulated from numerous transient transfections, making it unlikely that OR expression or protoglomerular targeting were influenced by positional effects related to where BACs inserted into the genome. We found that OSNs expressing a particular Gal4-modified OR locus nearly always projected to a single protoglomerulus. OSNs expressing different ORs from the same OR subfamily gene cluster were also found to project to the same protoglomerulus, and further, OSNs expressing ORs from different nearby clusters residing on the same BAC also projected to the same protoglomerulus.

**Fig. 9:**
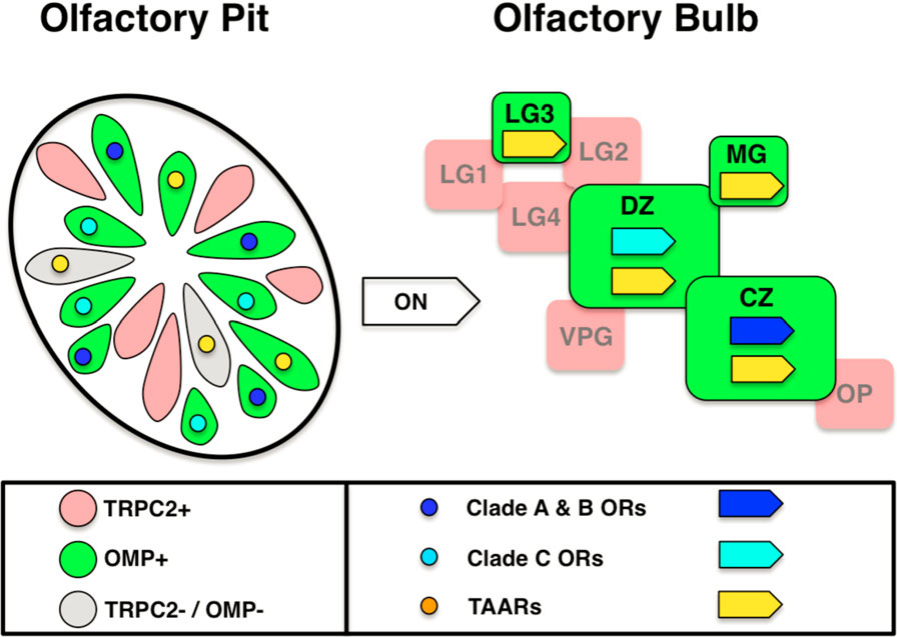
Organization of OSN projections to the Olfactory Bulb. TRPC2 expressing microvillous class OSNs (pink) express mainly V2R-like receptors and project to the LG1, LG2, LG4, VPL, and OP protoglomeruli (pink). OMP expressing ciliated class OSNs (green) express either traditional ORs (blues) or TAARs (yellow). OR expressing OSNs project to either the CZ or the DZ protoglomeruli, depending upon the specific OR they choose to express (Clades A,B: dark blue arrow; Clade C: light blue arrow). TAAR expressing OMP class neurons project to the CZ, DZ, LG3, and MG protoglomeruli (yellow arrows)

Since ORs are expressed in OMP class sensory neurons, we expected that OR expressing OSNs would project to the four protoglomeruli that are innervated by OMP class sensory neurons [23]. Surprisingly, we found that all of the ORs we sampled were expressed in OSNs that projected to either one of the two largest OMP class protoglomeruli: the CZ or DZ protoglomeruli. OSNs projecting to CZ chose to express receptors from OR clades A and B, comprising subfamilies or101 through or128, while DZ projecting OSNs chose to express receptors from OR clade C comprising subfamilies or129 through or137 (Fig. 9a). We infer that CZ and DZ are the major two protoglomerular targets of OR expressing OSNs. These results are consistent with the idea that OR choice and axon targeting are either tightly coordinated or dependent upon each other.

The formation of OR-specific glomeruli in the mature bulb and their reproducible positioning strongly suggest that OR choice and OSN axon guidance are not independent processes. Experiments in the mouse in which the coding sequence of one OR was replaced by the coding sequence of another revealed that the ORs themselves influence glomerular location [3, 10]. Recent work reported that the baseline G-protein coupled activity mediated by ORs regulates the expression of axonal guidance and adhesion molecules, providing a mechanism through which OR identity and glomerular position can be coupled [18]. Whether this mechanism contributes to the relationship we see between OR choice and initial axon targeting is unclear. Arguing strongly against this mechanism playing a role in targeting at this early stage is the observation that OSNs expressing many different ORs project to the same initial protoglomerular target. We observed that OSN axons projecting into the CZ protoglomerulus that express or111–7, or those that express or111–11, project to the whole CZ along the entire anterior-posterior axis (data not shown). We infer that protoglomeruli are not partitioned into separate areas at this early stage in development, and that the axons of OSNs expressing (in the aggregate) many different ORs are intermixed within protoglomeruli.

### OSNs that choose to express a TAAR project axons to multiple protoglomeruli

We also explored the possibility that some OMP class OSNs express TAARs that project to the remaining two OMP positive protoglomeruli that seemingly are not innervated by OSNs expressing traditional ORs. RNA-seq data from 2 dpf embryos suggested that TAARs are more highly expressed in OMP as compared to TRPC2 class OSNs. *In situ* hybridization with probes corresponding to each of three TAARs did not overlap with TRPC2 class OSNs. As predicted by the RNA-seq data, some of the OSNs expressing any one of these three TAARs were observed to co-express with an RFP transgene driven by an OMP promoter. These data suggest that at least some OMP class neurons express TAARs instead of ORs.

Surprisingly, our BAC data demonstrate that OSNs expressing any one of 3 separate Gal4-modified TAARs project to all four OMP class protoglomeruli, and less frequently to TRPC2 positive protoglomeruli as well. In mouse, TAAR expressing OSNs project to TAAR-specific glomeruli that are all located in a distinct region of the bulb [48, 49]. The more dispersed projection pattern of OSNs expressing TAARs that we observed in larval fish raises the question of whether they all ultimately converge upon single TAAR-specific glomeruli, or whether some of them send a more general signal to multiple glomeruli. Interestingly, one of the TAARs we examined, taar13c, has been identified as a high affinity receptor of cadaverine, a metabolite produced by decaying tissue [50]. Cadaverine induces a strong aversive response in adult zebrafish [50]. *taar13c* is expressed in adult ciliated OMP expressing OSNs [40]. This is in agreement with our RNA-seq data demonstrating TAAR expression in OMP positive sensory neurons. We also see *taar13c* expression in OMP positive OSNs by *in situ*, but the majority of *taar13c* expression at 3 dpf is in OSNs that do not express fluorescent tracers driven by either OMP or TRPC2 promoters. There are several possible explanations for the discrepancy between the essentially total overlap of* taar13c* and OMP expression in the adult as compared to the partial overlap we observe in larvae. For example, [1] our BAC-based mapping approach may be inaccurate, [2] the OMP promoter we used to drive RFP in our experiments may not be active in all ciliated sensory neurons, [3] *taar13c* expression may precede detectable OMP expression, or [4] *taar13c* expressing OSNs that do not express OMP may either suppress *taar13c* expression or die later in development. Brief exposure to cadaverine has been reported to induce the phosphorylation of ERK (Extracellular signal-Regulated Kinase) in the olfactory bulbs of adults. pERK was induced in the dorso-lateral region of the bulb and was most intense in a single glomerulus [40]. From these results it was inferred that *taar13c* expressing OSNs innervate a single glomerulus, and at higher cadaverine concentrations, additional OSNs are activated that express other TAARs and whose axons converge onto other glomeruli in the dorso-lateral region of the bulb. It was not possible to directly trace the axons of *taar13c* expressing OSNs in these previous experiments, so the evidence that *taar13c* expressing sensory axons converge into a single glomerulus was indirect. Our results at least raise the possibility that the more generalized activity evoked by cadaverine originated from *taar13c* expressing OSNs innervating more than a single glomerulus. Alternatively, the exuberant axonal projections we see early in development might be pruned back to a more limited set or eliminated by cell death.

### There is no segregation of OR expression in the early olfactory epithelium

There is a striking pattern of OR regionalization within the olfactory epithelia of mice. Early reports in the mouse suggested that 4 classes of ORs were expressed in well-defined territories in the nasal epithelium [51–54]. Subsequent studies suggested that ORs are expressed in overlapping bands of OSNs within the epithelium [14], [55], such that at any particular location OSNs stochastically express any one of a defined subset of ORs. One report of a similar pattern of overlapping bands of OR expression has been made in the adult zebrafish [56]. In the mouse, the axonal projection pattern of OSNs along the dorsal-ventral axis in the OB is related to their position along the dorsomedial to ventrolatetral axis in the olfactory epithelium [24], suggesting that pathfinding could be regulated by gradients of guidance receptor expression within OSNs in the olfactory epithelium that correspond to counter gradients of guidance cues expressed in the bulb [16, 57–60]. Our findings do not support a similar mechanism of initial guidance in the zebrafish. We have found that OR choice and axonal target location are tightly coordinated, but we failed to observe any obvious relationship between the OR an OSN chooses to express and its position within the olfactory epithelium. We did not detect distinct regions of the olfactory epithelium in which particular OR subfamilies are expressed, consistent with similar findings by Ngai et al. [61] in mature catfish and Barth et al. [41] in mature zebrafish. We did not observe the apparent banding of OR expression described by Weth et al. [56]. Since OR choice and axonal targeting are tightly linked, but OR choice and OSN location are not, our findings do not support a model of OSN guidance based on gradients of guidance receptors expressed in the olfactory epithelium.

We propose instead that the clade from which a sensory neuron chooses an OR to express and the expression of axonal guidance receptors are coordinated by common upstream transcription factors. At this early stage of development, OSN axons are guided to specific larger regions of the OB, for example, the protoglomeruli in zebrafish. Comparable regions may be more difficult to visualize in the mouse, but some regionalization is evident. Mouse ORs can be categorized as either fish-like (Class I) or terrestrial-like (Class II) based on sequence comparison [62]. Class I OR genes are confined to two adjacent sub-clusters on mouse chromosome 7 while Class II ORs populate ~40 gene clusters scattered over many chromosomes. The OB can be divided into two distinct regions based upon the projection patterns of OSNs expressing either Class I or Class II ORs [15, 63]. Bozza et al. identified a genomic element that can be used to drive expression of a marker in OSNs targeting the dorsal (Class I) region. A further distinct region is evident by the projections of TAAR expressing OSNs [49]. In both mouse and zebrafish, distinct axonal territories in the bulb are occupied by OSNs that have chosen an OR to express from a particular larger subset of ORs.

In *Drosophila*, some transcription factors have been shown to influence the choice of ORs and axonal targeting, suggesting a mechanism through which these processes could be coordinated [64–67]. Our working model is that OSNs have transcriptional mechanisms that coordinate the expression of axonal guidance receptors and the clade from which an OR is chosen. The guidance receptors that are selected through this process guide growing sensory axons to their initial targets, the protoglomeruli [23, 68]. OR-specific glomeruli emerge later in development by a process of coalescence from the larger protoglomerulus [19, 69]. We suggest that OR dependent processes that alter guidance receptor and adhesion molecule expression [18, 70, 71] drive the segregation of OR-specific glomeruli within these larger protoglomerular regions [69].

## Conclusions

We conclude that at early developmental times, even as OSNs are innervating the OB: [1] a significant proportion of the OR gene repertoire is expressed, [2] each OSN chooses a single OR to express, [3] neither OR choice or targeting are related at this early stage of development in any simple way to OSN location within the olfactory epithelium, [4] OR choice is coordinated with the expression of specific axonal guidance receptors, [5] through this mechanism OSNs project to specific locations in the OB where protoglomeruli form, [6] each protoglomerulus is targeted by OSNs that in the aggregate express many different ORs, and [7] OSN processes segregate into OR-specific glomeruli later in development. Our findings are not consistent with a model of axon guidance in which gradients of guidance receptor expression in the olfactory epithelium determine target location within the bulb as has been proposed along the dorsoventral axis in the OB of the mouse. Instead, they suggest that upstream transcriptional machinery coordinates OR choice and guidance receptor expression within OSNs.

## Abbreviations

CP: center of the olfactory pit
CZ: Central Zone
DZ: Dorsal Zone
ERK: Extracellular signal-Regulated Kinase
LG3: Lateral Glomeruli 3
MG: Medial Glomeruli
OB: olfactory Bulb
OR: Odorant Receptor
OSNs: Olfactory Sensory Neurons
TAARs: Trace Amine Associated Receptors
